# Unusual cholesterol crystal formation in a rare clinical case report of splenic echinococcal cyst in a patient from Sardinia, Italy

**DOI:** 10.3389/fpara.2024.1498099

**Published:** 2025-01-10

**Authors:** Cinzia Santucciu, Ashkan Hajjafari, Soheil Sadr, Scilla Mastrandrea, Carlo Rettaroli, Luca Simbula, Mariano Scaglione, Salvatore Masala, Angela Peruzzu, Giovanna Masala

**Affiliations:** ^1^ World Organisation for Animal Health (WOAH) and National Reference Laboratory for Echinococcosis, Istituto Zooprofilattico della Sardegna, Sassari, Italy; ^2^ Department of Pathobiology, Faculty of Veterinary Specialized Science, Science and Research Branch, Islamic Azad University, Tehran, Iran; ^3^ Department of Pathobiology, Faculty of Veterinary Medicine, Ferdowsi University of Mashhad, Mashhad, Iran; ^4^ Unità Operative Complesse (UOC) di Malattie Infettive, Azienda Ospedaliero Universitaria (AOU), Sassari, Italy; ^5^ Struttura Complessa (S.C.) Chirurgia Generale e d’Urgenza Azienda Ospedaliero Universitaria (AOU), Sassari, Italy; ^6^ Unità Operative Complesse (UOC) di Radiologia, Azienda Ospedaliero Universitaria (AOU), Sassari, Italy

**Keywords:** splenic cystic echinococcosis, *Echinococcus granulosus sensu lato*, cholesterol crystal, human diagnosis, case report

## Abstract

Cystic echinococcosis (CE) is a zoonotic disease caused by *Echinococcus granulosus* sensu lato, the metacestode of a tapeworm parasite of high medical importance. Infection of the parasite leads to the development of echinococcal cysts, and the spleen is a rarely infected organ. A 46-year-old woman who was born and who resides in Sardinia, Italy, was referred to the Echinococcosis outpatient clinic at the University Hospital of Sassari (Sardinia, Italy) for a pain in the left flank. She used to live in the countryside, in contact with several animals, and for 2 years, she had been working in a family garden, growing vegetables as a hobby. Ultrasounds and X-ray were performed, which evidenced a rounded formation in the upper third of the spleen, while a CT scan confirmed a parasitological cyst. Immunological examinations on serum samples did not detect specific antibodies against *Echinococcus* spp. Following surgical exportation, the whole spleen with the cystic lesion was delivered to the World Organisation for Animal Health (WOAH) and the National Reference Laboratory for Echinococcosis for further laboratory analyses. Moreover, characterization of the cyst fluid resulted dense and shiny. Observation under a light microscope at ×400 magnification revealed the formation of rectangular crystals and aggregates attributable to cholesterol molecules. Subsequently, through parasitological investigation, molecular biology investigations confirmed *E. granulosus* sensu stricto G1. We describe cholesterol crystals in a splenic echinococcal cyst for the first time. There is no clear explanation of how the cholesterol crystals formed in this case, but this was attributed to multifactorial causes, including atherosclerosis, chronic inflammation, parasite metabolism, and host responses.

## 1 Introduction

Cystic echinococcosis (CE) is a zoonotic disease, the causative agent of which is *Echinococcus granulosus* sensu lato (s.l.), the metacestode of a tapeworm parasite of high medical importance (genus *Echinococcus*, class Cestoda, family Taeniidae) (Eckert et al., 2001; Thompson, 2017). According to the recent “Global report on neglected tropical diseases” (Global report on neglected tropical diseases 2023, 2024), CE is among the 20 neglected diseases by the World Health Organization (WHO) whose public health burden is overlooked. CE is spread worldwide and is still present with varied frequency in hypoendemic, endemic, and hyperendemic areas. Sardinia, an island in the Mediterranean Basin, has the highest incidence (6.8/10^5^) (Craig et al., 2007; Brundu et al., 2014; Thompson, 2017).

The life cycle of *E. granulosus* s.l. comprises two species: the definitive hosts (canids) and the intermediate hosts (ungulates), mainly represented by sheep. Environmental contamination increases the risk of infection (Santucciu et al., 2023b), and humans are seldom infected, being dead-end hosts as they have no role in upholding the life cycle of the parasite (Eckert and Deplazes, 2004). When eggs are ingested, they hatch in the small intestine, and the oncosphere, a larval stage, can be relieved and go through the mucosa into the lymphatic and circulatory systems. Several organs can be infected by the parasite (Moro and Schantz, 2012). The oncosphere grows and gives origin to the metacestode stage, which gradually develops into echinococcal cysts, which can be asymptomatic for years (Moro and Schantz, 2012). The liver (75%) and the lungs (15%) are the first and the second filter, respectively. However, a low percentage (10%–15%) of embryos may spread into the circulatory system and reach abdominal organs such as the spleen.

Despite splenic CE having been first described in 1790 by Berlot after an incidental detection during an autopsy (Muro et al., 1969), the spleen can very rarely be an infection site (Humphreys and Johnston, 1979). The average global occurrence is less than 3%, as the worldwide incidence of splenic CE ranges from 0.5% in hypoendemic to 4% in hyperendemic areas (10 11). India and Iran are the most affected countries, with incidence rates exceeding 4% (Rasheed et al., 2013).

As in other organs, the detection of echinococcal cysts in splenic tissues is mainly an irregular finding, in addition to symptoms related to compression of the surrounding sites. This makes the diagnosis of CE in humans extremely challenging. The most reliable tools are imaging techniques. Ultrasound (US) is mainly used for abdominal cysts, whereas conventional radiography (X-ray) is the best option when there is chest involvement. Moreover, magnetic resonance imaging (MRI) and computed tomography (CT) are extremely useful tools in cases when more details are needed (Brunetti et al., 2010). Cyst classification has been set up according to viability: active (CE1 and CE2), transitional (CE3a and CE3b), and inactive cysts (CE4 and CE5). The stage and an early diagnosis are critical factors for the diagnosis, management, treatment, and follow-up of patients. However, imaging techniques are sometimes unsuccessful in providing a definite diagnosis. Therefore, immunological tests, such as immunoblotting (IB), enzyme-linked immunosorbent assay (ELISA), and immunochromatographic test (ICT), are generally used to complete and confirm CE diagnosis once immunoglobulin G (IgG) antibodies against *E. granulosus* spp. are detected (Peruzzu et al., 2022).

The pharmacological approach is the most utilized therapy, as well as in combination with other protocols, such as watch and wait, percutaneous treatment PAIR (puncture, aspiration, injection, re-aspiration), and surgery for echinococcal cyst removal (Brunetti et al., 2010). As a more invasive intervention, the latter is seldom employed and is only used as a life-saving treatment. Furthermore, investigations may be performed directly on the cyst. Parasitological examination leads to the detection of typical features of the cyst, while microscopic observation of the inner fluid confirms the presence or absence of protoscoleces to assess the fertility of the metacestode. Finally, biomolecular investigations, as the most reliable techniques, can definitely confirm CE disease (Brunetti et al., 2010).

This clinical report describes a rare case of a splenic cystic parasitic lesion confirmed as an echinococcal cyst. Direct inspection of the parasitic material revealed unusual crystal formations characterized as cholesterol aggregates, highlighting for the first time their occurrence in the spleen.

## 2 Case description

A 46-year-old woman who was born and who resides in Sardinia (Italy) was referred to the Echinococcosis outpatient clinic, Infectious Disease ward as, during a routine US abdominal investigation performed for a pain in the left flank, a cyst compatible with a parasitological formation was discovered in the spleen. A risk of rupture of the lesion was also immediately evidenced. A multidisciplinary approach was required to perform an early diagnosis and to correctly manage the patient.

Firstly, the patient filled out a questionnaire covering general information and any risk correlations (i.e., urban or rural habitation, dealings with animals, profession, habits, and travel history). It was found that she had no dogs and that she only owned a cat. Moreover, she used to live in the countryside and, for 2 years, had been working on a family garden, growing vegetables as a hobby.

During the review, the patient firstly referred to several details about her medical history: she suffers from pollen allergy, rhizarthrosis, and irritable bowel syndrome. Moreover, several years ago (2019) (**Table 1**), she did not live in Sardinia. By this time, she presented a discomfort on the left side; however, a US checkup showed a negative result. No other symptoms were reported.

**Table 1:** Timetable of the diagnostic process.

Date	Symptoms	Clinical investigation	Outcome
10/10/2019	Discomfort on the center side	Ultrasound	Negative
06/10/2023	Pain in the center flank	Ultrasound	Positive: round formation, echogenic content with a more solid component, finding compatible with an echinococcal cyst staged as CE4
31/10/2023	Routine visit	Ultrasound	Positive: round formation, echogenic content with a more solid component, finding compatible with an echinococcal cyst staged as CE4
26/01/2024	Pain in the center flank	Laparoscopic surgery	Positive: removal of the whole spleen with the cystic lesion

**Figure 1:**
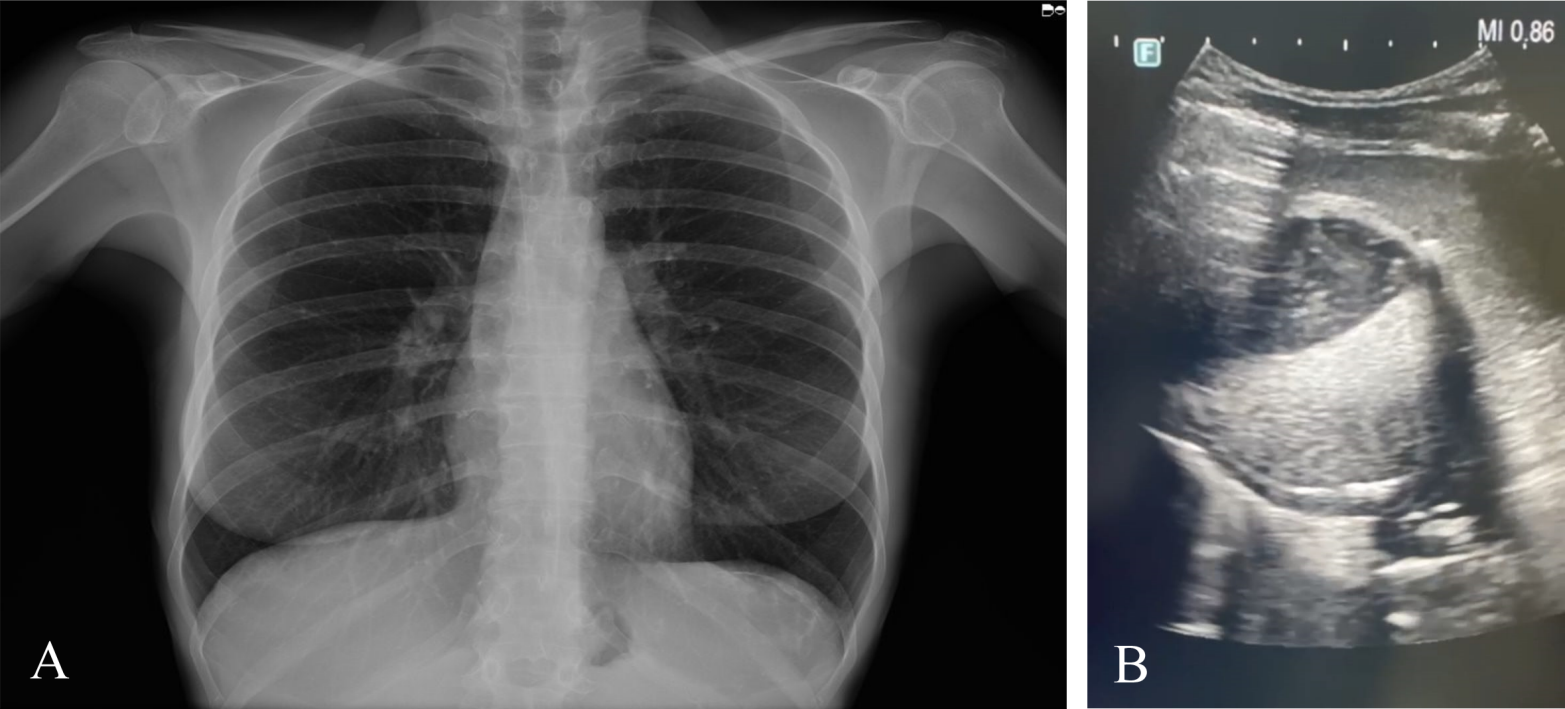
**(A)** Thoracic conventional radiography displaying an increased volume in the splenic area. **(B)** Ultrasound evidencing a round-shaped cystic formation in the spleen.

Thereafter, on October 2023, further investigations were performed in Sardinia (**Table 1**). Conventional X-ray evidenced an increased volume in the splenic area, while US displayed the presence of a rounded formation in the upper third of the spleen, in contact with the diaphragm, which measured approximately 8.0 × 6.5 cm (**Figure 1**). The lesion appeared expansive and partially calcified with dense fluid and corpuscular inner contents, as well as evident material floating inside. A finely echogenic content with a more solid component stratified in level was observed. The findings were compatible with a partially involute echinococcal cyst staged as CE4, according to the WHO (Working Group, 2003). A wall with irregular thickness was shown, from a calcified to a very subtle part.

Given the characteristics of the lesion, the patient was referred to an infectivologist for appropriate clinical management. To avoid any risk of cyst rupture, it was recommended to refrain from sporting activities and to avoid any effort. Moreover, more in-depth investigations were promptly prescribed to the patient: clinical examinations for additional details of the splenic lesion and blood analysis for general health status evaluation, which showed normal values. In addition, with the levels of transaminase and creatinine being normal and with serious suspicion of a parasitic lesion compatible with an echinococcal cyst, therapy with 400 mg (bis die) albendazole (ABZ, ZENTEL; GlaxoSmithKline, London, UK) for 3 months was promptly prescribed, in accordance with the guidelines (El-On, 2003). For the entire duration of the pharmacological treatment, blood chemistry tests were also performed every 30 days to monitor possible side effects related to ABZ.

Immunological examinations of the serum sample did not detect any specific antibodies against *Echinococcus* spp. (**Table 2**), either with the routine ELISA Echinococcus IgG kit (DRG, Instruments GmbH, Marburg, Germany) or with the Echinococcus Western Blot IgG (IB) (LDBIO, Diagnostics, Lyons, France) with higher performance.

**Table 2:** Patient data and radiological and serological findings.

Patient data			Radiological findings	Serological assay
Gender	Age (years)	Nationality	Cyst localization (stage)	ELISA/IB
Woman	46	Italian	Spleen (CE4)	Negative

*IB*, immunoblotting.

Thereafter, the patient was referred for surgical consultation for correct and rapid management (**Table 1**). All of the spleen had to be immediately removed given the characteristics of the cyst, i.e., very thin wall thickness and the significant size of the cystic formation filling more than two-thirds of the organ, in addition to the pressure exerted on the diaphragmatic organ. For this reason, the patient had to undergo a CT scan to reveal more in-depth details of the vascularization and interconnection of the splenic tissue with the surrounding organs, which is fundamental for the operation.

On admission to the surgery ward, the medical conditions showed normal general parameters: body temperature, 35.4°C; blood pressure, 121/78 mmHg; respiratory rate, 18 bpm; heart rate, 89 bpm; and oxygen saturation, 98%.

A laparoscopic surgery was performed for the entire duration of the intervention for removal of the whole spleen. This less invasive approach was used to reduce the infectious burden and to increase the chance of recovery of the patient. After full recovery, the patient was discharged on day 10 after the operation, with the ABZ pharmacological therapy still administered for 30 days (El-On, 2003). Her postoperative course was ordinary, apart from a pleural effusion that resolved in 2 weeks. Furthermore, follow-up assessments were performed first at 1 week and then at 1 month, thereafter every 6 months for a number of years, which will help determine complete recovery without signs of any recurrence.

**Figure 2:**
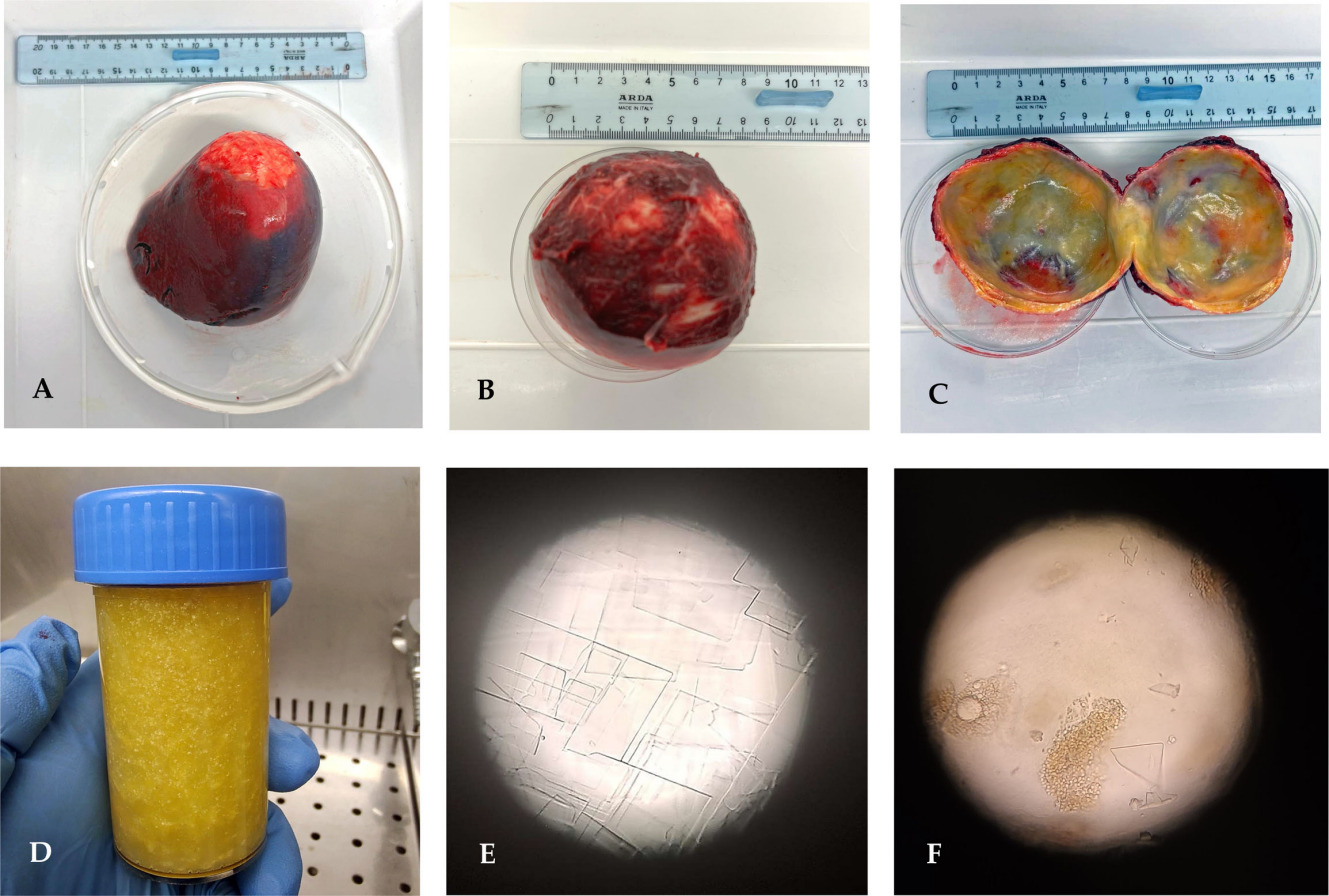
**(A)** Whole spleen harboring the echinococcal cyst. **(B)** Echinococcal cyst enucleated. **(C)** Internal appearance of the parasite wall. **(D)** Cystic fluid with dense and glittery features. **(E, F)** Light microscope observation of the cystic fluid presenting rectangular crystal formation and cholesterol aggregates (magnification, ×400).

The whole spleen with the cystic lesion (**Figure 2**) was delivered to the WOAH and the National Reference Laboratory for Echinococcosis for further laboratory analyses. The echinococcal cyst was isolated from the splenic parenchyma to allow parasitological examination (**Figure 2**). In the first step, inspection of the parasitic lesion with the naked eye was performed to identify pathognomonic signs of an echinococcal cyst. The presence of germinal laminated layers and cystic fluid was observed. Moreover, the cystic fluid was characterized as dense and shiny, and its observation under a light microscope (Zeiss, Axioplan) at ×400 magnification revealed the formation of rectangular crystals and aggregates attributable to cholesterol molecules. Any evidence of protoscoleces was described to assess the fertility of the cyst.

Subsequently, an aliquot of the cystic lesion was collected and submitted for DNA extraction as previously described (Santucciu et al., 2023a). Total genomic DNA was extracted using the DNeasy Blood and Tissue Kit (Qiagen, Hilden, Germany) according to the manufacturer’s instructions. The purity and the concentration were evaluated with the NanoPhotometer^®^ N120 (Implen GmbH, Munich, Germany). Finally, the species and genotype were determined (**Table 3**) by means of sequence analysis of two PCR amplification products: the mitochondrial genes *cox1* (Nakao et al., 2000) and *nad5* (Kinkar et al., 2018). The results confirmed the diagnosis of CE through the detection of *E. granulosus* s.s. identified as genotype G1 (**Table 3**).

**Table 3:** Parasitological and molecular findings.

Parasitological examination		Molecular analysis
Macroscopic observation	Microscopic observation	PCR *cox1/nad5* (*g*enotyping)
Parasitic features Cystic fluid of glittering appearance	Absence of protoscoleces Presence of cholesterol crystals	Positive for *E. granulosus* s.s. (G1)

## 3 Discussion

Medical and laboratory technologies have greatly advanced in recent years. However, the diagnosis of CE is still a challenge, which presents difficulty in determining whether a splenic cyst is parasitic or non-parasitic (Kang and Jeon, 2013; Kowe et al., 2015), until pathognomonic signs have been manifested as a result of the increase in size of the echinococcal cyst and the compression of the surrounding tissues.

In the present report, we described a clinical case of a rare CE in a female patient from Sardinia, Italy. Clinical and laboratory investigations led to the identification of the presence of an unusual splenic echinococcal cystic formation. After direct inspection of the parasitic material, uncommon crystal formations were observed, to our knowledge, for the first time in the spleen.

In this specific case report, the surgical removal of the cyst, along with the pharmacological treatment, was the only life-saving option as the cyst presented a risk of rupture. Other modes of treatment include watch and wait and percutaneous treatment PAIR.

Nevertheless, in spite of the several difficulties encountered in this study with regard to the diagnosis of splenic echinococcosis disease, the pathognomonic signs were observed (Mansour et al., 2023), followed by a suspicion from the US results and a confirmation by CT. US is generally described as the most reliable technique for CE abdominal involvement; however, in this particular and rare case of splenic lesion, the first examination showed a negative outcome. Conversely, the final US analysis addressed for a parasitological diagnosis since evidenced a partially calcified lesion with dense fluid, corpuscular inner contents, floating material, and echogenic content. This confirms that only an expert and a trained physician is able to recognize the typical features of an echinococcal cyst, such as the hydatid sand and the double line sign represented by the two layers of the pericystic wall and the laminated membrane (Czermak et al., 2001). In addition, the serological examination did not confirm any presence of specific antibodies against *Echinococcus* spp., making the diagnosis more complicated. However, this occurrence can be due to the immunodiagnostic tests being influenced by several cyst-related characteristics, such as the number, stage, size, and location. As previously reported, the involvement of organs different from the liver, such as the lung, kidney, or spleen, is linked to lower levels of specific *Echinococcus* spp. antibody in human sera (Zahoor et al., [[NoYear]]; Zhang et al., 2012). Moreover, one of the major challenges in CE diagnosis is the need to perform a differential diagnosis to exclude neoplastic lesions or abscesses and to confirm an infectious disease (Eckert et al., 2001; Krige and Beckingham, 2001). Hence, a multidisciplinary approach is finally essential to provide an early diagnosis for the correct management of the patient. Along with CT, direct investigation of the echinococcal cyst and molecular analysis confirmed the parasitological source of the lesion by identifying *E. granulosus* s.s. genotype G1 as the etiologic agent, with G1 being the most represented genotype in Sardinia, as evidenced by our previous investigations (Santucciu et al., 2023a).

Interestingly, direct examination of the cyst by parasitological analysis showed the presence of an extremely uncommon cystic liquid of gold color, shiny and opaque, that, when observed under the microscope, revealed the formation of irregular quadrangular crystals of cholesterol. This is an extremely uncommon condition, despite having been rarely described in other organs, as it has not been described before in the spleen. Similar findings have been described in a few case studies. The gallbladder and the arterial plaques are the most commonly associated with the formation of cholesterol crystals (Portincasa et al., 2023).

There is no clear explanation for the origin of the cholesterol crystals in this case. Several factors could have contributed to the formation of these crystals, many of which are metabolic and chronic issues and could also involve host tissue interaction with parasites (Fallah et al., 2021). However, multifactorial causes, including chronic inflammation, parasite metabolism, and host responses, may be evaluated. Moreover, the long-term presence of the cyst creates a microenvironment favorable to cholesterol precipitation from the cystic fluid (Waitupu et al., 2024). Hence, as a consequence of these findings, the cyst had likely been present for a considerable time, which allowed cholesterol to accumulate and crystallize (Baumer et al., 2021).

Several characteristics distinguish cholesterol crystals from others, such as their density and high shine observed macroscopically, coupled with the rectangular crystallizations seen under microscopic inspection (Varsano et al., 2022). Echinococcal cysts may be associated with cholesterol crystals, affecting their diagnosis and treatment. Although echinococcosis is primarily caused by parasitic infection, cholesterol crystals can indicate a secondary process that might affect clinical management. They could complicate surgical removal or adversely affect postoperative recovery by contributing to local inflammation and fibrosis (Sadr et al., 2022). Atherosclerosis is also one of the most well-documented chronic inflammatory conditions that produce cholesterol crystals (Waitupu et al., 2024).

An interesting study on hepatic CE in sheep described the biochemistry and showed fertile lesions containing several minerals despite the low levels of cholesterol, lipids, protein, and glucose (Hasona et al., 2017). The source of cholesterol in these cysts had been speculated to be *Echinococcus* as it appears incapable of producing *de novo* lipid synthesis; therefore, it takes its lipids from its host in order to provide it with the necessary lipids to sustain life and form cyst walls (Romano et al., 2015).

An earlier study reported that the parasite’s AgB secretion carries the host lipids needed for parasite metabolism while intervening in the host immune response through its immunomodulatory properties. Similar studies also confirmed the absorption of cholesterol into echinococcosis cysts (Silva-Álvarez et al., 2016).

Furthermore, another study demonstrated that the cholesterol crystals in the parasitic cyst fluid are significantly associated with the decreased viability of protoscoleces (Shaheen et al., 2022). This suggests the immunomodulatory role of cholesterol through the nitric oxide pathway. A decreased viability of protoscoleces is associated with high inducible nitric oxide synthase (iNOS) expression in cyst walls, which contain cholesterol crystals. In light of these findings, cholesterol plays an important role in the immunopathology of hydatid disease, emphasizing that cholesterol might also have a role to play in cyst degeneration.

Finally, a wide point of view is necessary in hyperendemic areas such as Sardinia in order to decrease several risk factors. Through analysis of the questionnaire submitted by the patient, it was discovered that she had been working in a family garden and growing vegetables as a hobby, which resulted into contact with raw vegetables and with farm animals. Hence, only correct management could prevent sustaining the biological cycle of the parasite, such as by washing the vegetables and the hands appropriately before eating (Santucciu et al., 2023b; Umhang et al., 2024).

## 4 Conclusions

The findings presented in this rare clinical case allowed concluding a positive diagnosis for CE in the patient due to the presence of a splenic echinococcal cyst, despite several examinations showing negative results. Hence, the etiologic agent was finally confirmed as *E. granulosus* s.s. G1. To our knowledge, this report demonstrated for the first time the formation of cholesterol crystals into a splenic cyst. Similar findings have been described in a few case studies in the literature, but only in other organs. Moreover, it can be concluded that multifactorial causes comprise the basis of cholesterol crystal formation, including chronic inflammation, parasite metabolism, host responses, and atherosclerosis, along with time, which has to be considerable to allow cholesterol to accumulate and crystallize.

## Data Availability

The original contributions presented in the study are included in the article/supplementary material. Further inquiries can be directed to the corresponding author.
